# Problematizing “Activism”: Medical Volunteer Tourism in Central America, Local Resistance, and Academic Activism

**DOI:** 10.1177/1940844720948066

**Published:** 2021-11

**Authors:** Phiona Stanley

**Affiliations:** 1 3121 The Business School, Edinburgh Napier University, UK

**Keywords:** activism, volunteer tourism, critical medical ethics, homophobia, postcolonial theory, resistance

## Abstract

This paper critically examines epistemological, ontological, and axiological tensions of activism in three related contexts. These are, first, (primarily medical) volunteer tourism ideologies and practices in Central America, including U.S.-American teenagers volunteering in medical centers where, entirely untrained, they do sutures and injections, deliver babies, and help with amputations. Second, the paper considers and critiques local norms (e.g., widespread homophobia) and materials (e.g., the use of short-handled agricultural hoes) that may be discursively constructed as resistance to western imperialism. Finally, the critique turns back on the researcher gaze itself, problematizing the notion of academic activism in spaces, like these, where criticality itself is an imported—arguably luxurious—folly. Local people, it is apparent, do not want convoluted theorizing or Western hand-wringing; they want proper medical care. The paper therefore considers the extent to which academic work in such spaces can call itself activism at all. Three years of ethnographic research inform the paper (2013–2015, predominantly in Guatemala and Nicaragua), including hundreds of hours of interviews and participant observational fieldwork, in Spanish and English, with local stakeholders (e.g., teachers and homestay hosts) and Western volunteer tourists. The paper is theorized with reference to postcolonial theory, critical medical ethics, and liberation theology.

## Introduction

When can a film be considered obscene? And/or when is banning that film an infringement of people’s rights? This matters in the Unites States, as the First Amendment explicitly protects the freedom of speech. But if these rights are to be upheld, a semantic boundary needs to be drawn around obscenity, determining precisely which freedoms are, and are not, protected. It was for this reason, in 1964, that the Supreme Court ruled on the *Jacobellis vs. Ohio* case. Nico Jacobellis, a cinema manager, had been fined for showing the Louis Malle film *Les Amants*; the State of Ohio had deemed the film obscene. But was it? And, if so, how was “obscenity” to be defined? Previously, the Supreme Court had upheld a deductive ruling (in the 1957, *Roth vs. United States* case), defining obscenity as material where the “dominant theme, taken as a whole, appeals to the prurient interest” of the “average person, applying contemporary community standards.” (And later, in 1973 *Miller vs. California*, it would overturn the *Jacobellis vs. Ohio* ruling with another deductive definition.) But the 1964 case was different—and intellectually of great interest—as it relied on *inductive* (or, arguably, *abductive*; Mingers, 2012) rather than *deductive* reasoning. In the 1964 case, Justice Potter Stewart ruled:

[U]nder the First and Fourteenth Amendments, criminal laws in this area are constitutionally limited to hard-core pornography. I shall not today attempt further to define the kinds of material I understand to be embraced within that shorthand description, and perhaps I could never succeed in intelligibly doing so. But I know it when I see it, and the motion picture [*Les Amants*] involved in this case is not that. (Justia, 1964)

Stewart’s line, now famous, “I know it when I see it,” applied in this case to pornography. But might it equally apply to *activism* as used in both popular and academic discourses. What *is* activism? Can we define it? Or is it just that we “know it when we see it”?

In problematizing the definition of activism, this paper presents three linked cases in which self-professed activists each claim to be doing activism. At issue is the fact that their activisms are contradictory in both their goals and their effects. Logically, then, not all can be *doing* activism, if activism is definable by its aims and/or its effects. But perhaps it is not. Perhaps it is enough to have goodwill—to be “helping”—however vaguely conceptualized. (Although, see Tilley-Lubbs [2016]) for a critique of well-intentioned activism that had deleterious effects.) The three cases described in this article thus present a quandary. If all *are* examples of activism, it is necessary to trouble the notion of what activism actually is. Can it be defined by its aims? Its effects? And/or a vague sense of goodwill? Or is it necessary to rely on an inductive definition: that we know it when we see it? This is how the term seems to be used in both popular discourse and also in scholarly accounts. For example, Mendes (2015) studied feminist activism, Boykoff (2014) studied Olympics-protest activism, Kirshner (2015) studied youth activism, Carter (2018) studied environmental activism, and Reynolds and Cohen (2016) studied urban farming as activism, and yet none troubled the construct of activism itself.

But if we rely on an inductive construction—the assumption that *we know activism when we see it*—it is also necessary to trouble the “we” that is doing the perceiving. Does activism necessarily invoke a progressive politics, and, if so, how is this to be defined? Or is it still “activism” if it is the alt-right that perceives the objective as desirable social change? Can we, for example, call it “activism” when Swedish far-right organizations spread hate on YouTube (e.g., Farmer, 2005)? Can we describe as “activists” those who participate in Ku Klux Klan and neo-Nazi groups in the United States, including an anti-Jewish movement called “SS Warriors” (Blee, 2016)? And how might we describe the English Defense League, an organization mobilized “around a core narrative of the threat posed by ‘militant Islam’ to the UK and more generally the west” (Busher, 2013)? In each of the scholarly accounts, these groups *are* termed “activists.” But if any and all social-change goals are the legitimate target of “activism,” does the term retreat from usefulness, becoming a catch-all for any and all impassioned or vaguely “helpful” activities—per the paradigm of the in-group itself—that can be wrapped up in “activism” to confer legitimacy?

This paper teases out these theoretical issues through the following examples. First, I examine foreign volunteer workers in Central America. Second, I consider local people’s *resistencia* [resistance] to what they perceive as foreigners ignorantly interfering in local practices. In particular, I focus on untrained “nonmedics” (section titled “Nonmedics”), contested values (section titled “Values”), and the short-handled agricultural hoe (section titled “*El Cortito*”). These are contested spaces offering rich conceptual pickings. Against a potent, recent, and very bloody history of U.S. cultural imperialism in Guatemala, western sojourners and their local hosts array themselves along a continuum of positions, from wishing to invite/impose U.S. norms and practices (under rubrics of “helping” and “expertise”) to subscribing to (agri)cultural relativism and not wishing to exacerbate axiological violence through the adoption/imposition of foreign ways. Finally, I briefly consider academic activism (section titled “Academia”), and the capacity—or otherwise—of academic work to effect change.

Before this is possible though, it is necessary to examine the postcolonial historical context (in the following section) and volunteer tourism—sometimes referred to in its portmanteau form, *voluntourism*—both as a phenomenon and as a discourse community (section titled “Volunteers”). It is also worth noting that this conceptual paper is not intended as an ethnography in its own right. Instead, it draws upon interpretive data already presented and analyzed in a book-length study (Stanley, 2017) for the purpose of *problematizing tensions and issues within activism*. For this reason, a complete methodological account is not repeated here. In brief, though, the study from which the data excerpts are drawn was based on 19 weeks of participant research over a 3-year period (2013–2015) among Western sojourners engaging in volunteer projects, learning Spanish, and/or staying in host families in Guatemala and Nicaragua. One hundred and twenty people participated directly in the study—in interviews and focus groups—including NGO directors, teachers, homestay hosts, Spanish-language students, and volunteer workers. The nationality breakdown of the interviewees was as follows: 63 U.S.-Americans, 27 Guatemalans, 14 Europeans (from the UK, Netherlands, Denmark, Italy, Germany, and Switzerland), eight Nicaraguans, five Canadians, and three Australians. In total, the study was based on 104 hr of interview recordings and 407 pages of field notes. Its data were coded and analyzed inductively, following the principles of constructivist grounded theory (Charmaz, 2014).

## History

Everything in Latin America starts with history and politics. And ancient and modern, these are histories covered in blood:

Latin America is the region of open veins. Everything, from discovery until our times, has always been transmuted into European—or later United States—capital, and as such has accumulated in distant centers of power. Everything: the soil, its fruits and its mineral-rich depths, the people and their capacity to work and to consume, natural resources and human resources. Production methods and class structure have been successively determined from outside…always for the benefit of the foreign metropolis of the moment. (Galeano, 1971, p. 2)

Five hundred years after the sinking of European teeth into Indigenous throats, Latin America still lives the open veins of colonization. So much blood. So much death.

First, there was the Spanish conquest, and then, for hundreds of years, there was exploitation. Independence came, but by the 1920s, all that had changed was the master (Booth et al., 2014). By 1930, the United Fruit Company was the largest employer in Central America and the biggest landowner in Guatemala. From 1944, 10 years of “springtime” came under land-reforming presidents Arévalo and Árbenz. Unused United Fruit Company lands were nationalized, and workers gained a few rights. But the United Fruit Company, friends with the U.S. government, felt aggrieved. And to the United States, this looked way too much like a worker’s revolution. So, the CIA overthrew Árbenz and installed a military dictator. And over the next 40 years, 200,000 people were massacred, the majority of them Indigenous, Mayan civilians killed by government paramilitaries. More blood. More death (Grandin et al., 2011).

Nicaragua’s story is slightly different, but it starts the same: the *conquista* [conquest] and foreign exploitation. But then, in the 1930s and again in 1979, the people’s protests worked, to some extent. Again, they rose up against a U.S.-funded puppet and even though the Sandinistas—the Nicaraguan revolutionaries—celebrated victory in 1979, the civil war dragged on through the 1980s, killing about 30,000 people. More blood. More death (Wade & Walker, 2011).

But even through the civil war, the *Sandinista* government improved literacy, health care, workers’ rights, and education, and they did this, in part, thanks to the *brigadas internacionalistas*, the international brigades. Foreign doctors, teachers, engineers, and agriculturalists came, this time to help. They came from Europe, the United States, and elsewhere in Latin America: Che Guavara was an *internacionalista*, a medical doctor from Argentina who helped the revolution in Guatemala in 1954 before moving on to Cuba, the Congo, and (finally, fatally) Bolivia. So, there’s a history, here, of international helpers (Wade & Walker, 2011).

## Volunteers

Western volunteers still come. Nowadays, they talk about “doing development” or “aid work” rather than social justice or overthrowing tyrants. They don’t talk about liberation theology (e.g., Gutiérrez, 1973) or structural violence (after Galtung, 1969) in the *gringo* bars of Central America, like they did in the 1980s (e.g., Unferth, 2011). Now, they channel Angelina Jolie and the white-savior optics of *Instagram*, blaming bad luck and corrupt local politicians, blind to their own complicity in an international system of greed. The volunteers describe “helping out,” as if their presence—a few crumbs from their table—can change a global economic system designed and perfected in their favor. Let us not forget:

[*T*]he poor person does not exist as an inescapable fact of destiny. His or her existence is not politically neutral and is not ethically innocent. The poor are a by-product of the system in which we live and for which we are responsible. They are marginalized by our social and cultural world. They are the oppressed, exploited proletariat, robbed of the fruit of their labor and despoiled of their humanity. Hence the poverty of the poor is not a call to generous relief action, but a demand that we go and build a different social order. (Gutiérrez, 2004, p. 44)

But the Westerners don’t call themselves *brigadistas* anymore. Now, they are *voluntourists*, or just tourists, often backpackers, and sometimes students of Spanish, usually staying in homestays, and sometimes learning local crafts. And just as Central America provided bananas, because that’s what the international system wanted, now it provides voluntourism. In developing-world playgrounds, young Westerners play identity games, reinscribing old colonial relationships. Instead of extracting cotton, sugar, and slaves, the colonizer now extracts character-building challenges on which young people might cut their teeth. Otherwise, little has changed.

Whole industries have sprung up in Central America selling formative experiences to young *gringos*. Goudge (2003, p.35) writes:

[Volunteer work provides] a chance to improve one’s career prospects by cutting one’s teeth on ‘unusual’ challenges.…This is a continuation in a different guise of the old colonial relationship whereby colonies were regarded as essential providers of what the ‘mother country’ needed and desired. Only in those days it was slaves, sugar and cotton rather than character-building safari adventures.

The volunteers are still changing lives, but now it’s mainly their own. This is to say that a codependent relationship still exists, in which Central American tourism accommodates longer-term sojourners engaged in various activities that, arguably, serve the needs of the sojourners more than they do the local people. But the tourism industry matters enormously to local economies, too. Tourism contributes 9.1% of Nicaraguan gross national product, for example, and provides almost 200,000 people’s livelihoods (World Travel and Tourism Council, 2014). This codependency is thus still one in which power is tilted heavily in favor of those from outside, and as Galeano argues (above), Latin America is (still) a region of open veins, its resources transmuted into, determined from, and accumulated in distant centers of power for the benefit of the foreign metropolis of the moment.

But this is not the discourse within which volunteer tourism is undertaken. Instead, social imaginaries of “helping,” “authenticity,” and “fun” permeate volunteers’ own descriptions of their activities and also the advertising that allows them to find opportunities. All over the *gringo* press in Central America, you see ads in English for volunteer jobs. Here’s one: “Weekend staff at Black Cat Hostel. Free food, cheap drinks. 3 months minimum commitment. … Evening and weekend bar staff needed at the Old School Bar. Guaranteed fun. Give José a call” (*Xela Who Magazine*, June 2015). Here is another: “Put your education to use by teaching at the Community Center! … support education [for] over 200 kids in need in the community” (*Kamalbe* poster, shown in Stanley, 2017, p. 97). Other ads offer volunteer projects in coffee farms, kindergartens, and even a domestic violence shelter. Why, I ask the *gringos* working here, why do they think people hire them, the *gringos* rather than the locals? And one tells me:

I got a job as a waitress. I joined a band. Like, either one I wasn’t qualified to do in the US.…Here, I literally just walk up to people and [I’ll] be like, ‘Hey I think you need a waitress’, ‘alright’, ‘good, that’s me’.…I’m also very go getter, yeah, and I’m very active and have a lot of ideas. So maybe that’s partially just me. I think, yeah, there’s just a lot of opportunities to start working on projects [here].…I think [local people] could [do the same] if they wanted to, yeah. (A participant—pseudonym “Amber”—cited by Stanley, 2017, p. 2)

Let us be clear. This volunteer cannot do what Che Guevara did; she’s not a doctor. But she *is* go-getting and active, and she has lots of good ideas. Local people *could* do this too, if they wanted to. *If they wanted to*. The problem is, seemingly, that they don’t have good ideas and they’re not go-getter enough. And also: they can’t afford to work for free. And perhaps volunteer tourists’ Whiteness confers on a bar or a band the cachet of *gringo* approval.

But is this participant uniquely blind to her own positioning? Should we blame her? I don’t think so. Along with so many others, this participant is part of a wider discourse in which “helping” irreducibly constructs the “helped” as rather helpless. This is well documented in critiques of “development” (e.g., Farmer, 2005) and is a position that permeated the narratives of almost all the volunteers interviewed for this study. Am I therefore shooting fish in a barrel by citing and then critiquing these participants, embedded as they are in a paradigm of which I am critical? Do I seduce and betray them, building trust and then breaking it down as I re-present them as nasty imperialists? I hope not. My view is that *they are not to blame*. Instead, they are part of a *system* that is to blame, and also one in which we are all complicit, particularly those of us who work in center-west education. As long as our medical schools value prequalification experience of dubious ethical standing, premed students will seek out opportunities to practice on human beings in the majority world (Stanley, 2017, pp. 114–120). And as long as employers and colleges value (and in some cases, even *promote* and even *give credit for*) volunteer tourism, it will thrive. So, when their quotes are read from outside the volunteer-tourism discourse community (as I and presumably some readers are doing), the participants sound naïve (at best) and actively harmful and neoimperialist (at worst). But then does critiquing their discourses fall into precisely the trap of “academic activism” described in the “Academia” section? Is there a risk, here, of axiological and epistemological imperialism of our own, in which our morally superior, more “woke” discourses trump those of naïve volunteer tourists who are gamely trying to “help”? Herein is part of the paradox that *even this paper itself* may problematically be part of academic activism. This discussion continues in the “Academia” section.

## Nonmedics

But some voluntourism is even more harmful and here—I’m sorry—we come back to blood:

I was talking to someone who was in the process of applying to medical school, and he was like, ‘You’re going on an international volunteer trip for medicine?’ I said, ‘Yeah, I’m really excited’. He was like, ‘Yeah, I helped deliver a baby my first day [in Guatemala]. I literally landed and I went to the clinic and I did that. Then I performed a pap smear, too’. A man, an American male, doing that to …a Guatemalan woman. There’s just so many different …lines that are crossed and a lack of understanding between those two parties. (Nadia, early twenties, USA, cited by Stanley, 2017, p. 115)

The first week [in the clinic in Masaya, Nicaragua] was even more intimidating because Spanish was so difficult because they talk so fast, and it’s all so chaotic.…They assumed that we were doctors. So, I guess they thought we were stupid.…To work there you had to put in a donation [of US$300/month]. So, I’m not really burdening anyone because I’m, I already helped. So, it’s not a big deal if I’m an idiot.…Some of the nurses are a lot more welcoming, and they let me do some injections.…[As] a volunteer, I guess, you do whatever you can to help. And I think I’ve helped. (George, early twenties, USA, cited by Stanley, 2017, p. 80)

All the medics around the table…describe the [Nicaraguan] clinic/hospital as shocking, insufficient,etc. Blood all over the floor, gloves/instruments reused between patients. [One of the NGO coordinators] Danny told me [earlier that day] about scissors so blunt that to cut suture thread you have to pull at the stitches a bit, and the wound sometimes then reopens. In this case, they got the scissors from the clinic [and] sharpened them. [They also] bought the clinic a suture kit with proper surgical scissors. [The clinic] had been using the folding scissors you get in a sewing kit. Later [over dinner], Candace [one of the volunteers, aged 19, no medical training whatsoever] says she wants to do skydiving in the USA when she gets home. Holly [one of the NGO staff] tells her, ‘You can do it in San Juan del Sur [Nicaragua]’.

‘No, no way. Nica is way too sketchy’, Candace says. Nicaragua as ‘sketchy’ is based on her experiences at the clinic.…Having come thinking she was helping a terribly poor country, so very poor that it needed her as a medic, has she now had this confirmed? Nicaragua is so very poor, so ‘sketchy’ that it cannot do anything ‘properly’, whether skydiving or suturing. (Field notes on a focus group, Stanley, 2017, pp. 115–116)

This is the dark side of what Ibarra and Petriglieri (2010, p. 10) call “identity play,” defined as engagement in the provisional but active trial of possible future selves. While Candace, Amber, George, and others try out future identities in majority-world playgrounds, the experience is far from playful for their patients, whose lives and dignity are at stake. Instead of *learning from* Central Americans, the voluntourists are *practicing on* them.

## Values

There’s another problem with voluntourism, and it’s one of positionality and the reinscribing of colonial-type power relations. Goudge (2003, p. 17) says:

[*T*]he constant flow of development visitors from the materially better off countries…[may] unwittingly transmit a message that [practices] in the West are superior to those in [the South]…The message of superiority and corresponding inferiority, repeated endlessly in relation to all aspects of life, contributes to people believing the idea that everything in the West is superior[.]

This is why Candace and George are able to *perform* as doctors, because local people expect the *gringos* to know better, even when—as in this case—they don’t.

But what happens when *gringos do* know better? What happens if the West *is* getting something right? One issue that came up often in the data is that of deeply entrenched homophobia and transphobia in Central America:

There are instances [in my Spanish class, in Xela, Guatemala]…where I’m like, ‘I do not believe that’. For instance, talking with a teacher who…was talking about [her] view on gay people. That’s something that I found really hard to deal with.…It’s just something where her core belief…was that people who are homosexual were incorrect, like there was something wrong with them, that there’s a disorder, when I know that’s not true. They say that…homosexuals will go to hell … She was explaining that she thinks there’s a spirit, like a demon-ish spirit controlling them. That just contradicts what I know. (Amy, early twenties, USA, cited by Stanley, 2017, p. 90)

I’ve had experiences with some of my [local, Spanish-language] teachers [in the western highlands of Guatemala]…When they’ve asked about my romantic life, and I am [a woman] married to a woman.…On Monday I actually had to ask to change teachers because [my teacher’s] reaction was so awful.…He said, ‘I’m fine with that’, and then went on to equate homosexuality with prostitution, to say that transvestites were members of gangs and that people were right to be scared of them because they were like these big tattooed men in women’s clothes. (Alice, mid-twenties, UK, cited by Stanley, 2017, p. 89)

There is a tension here. On the one hand, there *are*—or should be—certain universal values, such as a respect for all human beings, and therefore an acceptance of LGBTIQA+ identities. I went to a small but vocal *Pride* parade in Xela, Guatemala, in 2015 and waved a rainbow flag. But I also ducked a confrontation when my homestay host in Antigua, Guatemala, was openly homophobic:

I was staying with a host family and [my host mother], over a few drinks on the Friday night, was really homophobic. Like wildly homophobic, equating people who are gay with child molesting and pedophilia and stuff. I was like, ‘Um, some of my best friends –’ but she wouldn’t hear it. So, I made an excuse and went to bed early because I thought, ‘I don’t want to sit and drink with this woman, but also I don’t want to rock this boat, and I’ve got to stay here’. But I felt so guilty afterwards for not saying anything. … Should I be standing up for people [for example, my close friends who are gay] who aren’t there? I was just hiding behind [seeming] straight privilege. I had talked about my ex-*novio* [ex-boyfriend].…Then someone said, ‘Oh no, no. Choose your battles. You’re not going to change that woman’s mind. You can’t go in being all colonial. It’s not your place’. (Stanley, 2017, p. 128)

I wonder about my own activism: was I an activist (or an imperialist?) when I attended the Pride parade? And was a cop-out (or was I decolonizing tourism?) when I chose not to engage with my host’s homophobia? It is easy to frame either course of action as activism, or not. I might ask: who am I to tell Guatemalans what to think? But I might equally ask: where was my calling out of social injustices? Neither question can be adequately resolved within the vague rubric of “activism.”

Some interviewees grappled with the same issue:

[The Danish volunteers, in Granada, Nicaragua] did a sexual education class a couple of weeks ago.…For the [high-school] kids to be in our program, the Nica[raguan] parents have to be accepting of what we’re teaching them.…When we’re teaching tolerance for homosexuality…because there is a deeply, deeply embedded homophobic culture [in Nicaragua].…I think [ours] is just a very progressive approach to education. In a lot of our home universities, our schools, it wouldn’t be seen that way, but compared to the culture here, it is. (Sally, mid-twenties, USA, cited by Stanley, 2017, p. 100)

To Sally, the power dynamics of these competing values are simple: tolerance is the Trojan horse invited in with the free, after-school program run by Westerners.

But to Sam, the issue is more nuanced, especially in light of the bloody history of imperialist meddling, not least by the United States, in Central America:

I don’t feel like you can go into a place [in Sam’s case, Quetzaltenango, Guatemala] that’s not yours, where you’re a foreigner and where your passport gives you incredible privilege…and your country has a terrible legacy—I really don’t feel like you can go into a place and lecture people. Even though I feel uncomfortable, after a while [of] my [host] family [saying], ‘You need a *novia*’ [girlfriend]…at the same time, I still don’t feel like I’m in a position where I can be like, ‘Okay, you need to shape up’. …As much as I would love to see a flowering of LGBT acceptance in Central America…I think it’s kind of the white man’s burden, and is it the *gay* white man’s burden if I come in and lecture…the people I interact with on a daily basis to say, ‘Okay, this is my identity and my identity’s getting accepted in my country and you need to accept it’? (Sam, early twenties, USA, cited by Stanley, 2017, p. 94)

## *El Cortito* [the short-handled hoe]

The medical voluntourists say *they* are doing activism, the Danish volunteers say *they* are doing activism, I say *I* am doing activism. Are we *all* activists? None of us? Some of us? Defined how? I tried resolving this swirl of questions by asking local people. Chatting about my research with cab drivers and homestay hosts and other strangers–I told them about the nonmedics and the problematics of power, explaining it simply, in Spanish, as something I was curious about, and asking what they thought—most shrugged and said, “*Pues, nos ayudan*” [Well, they’re helping us]. Implicitly, they mimicked the *internacionalistas*' [international brigades'] discourses of the 1980s, seemingly grateful for the table crumbs however inadequate. And then I asked myself: was my shiny criticality a form of imperialism, too?

Then a Guatemalan teacher, critical and forthright, told me about local resistance to the voluntourist antics of the *gringos*, especially those instigating projects of their own devising. Framing local people as opportunistic but ultimately savvier than the *gringos*, who “didn’t know any better,” Barbara inverts the trope of foreign aid workers leading hapless peasants out of ignorance. Her voice is gleeful as she recounts:

*Gringos* came to the countryside [in the western highlands of Guatemala, near Lake Atitlán] and said, ‘Oh my God, they will *ruin* their backs weeding with those short-handled hoes! Don’t they realize?’ So, they went back to the USA and raised money for the poor, ignorant Guatemalans. And, proudly, they brought back their long-handled hoes, showed people how to use them, and handed them over. The local people were very happy. Lots of nice, smiley photos were taken. These were really good hoes with heads made from 1 single piece of reinforced steel. They were much better than the cheap, Chinese-made ones from the local *ferretería* [ironmonger]*,* which tended to break where the two pieces had been welded together. When the *gringos* left, the people cut the handles down to the length they were used to, because that’s what they were used to and what they knew how to use. No one thought anything much of it. The *gringos* just didn’t know any better. (Barbara, early fifties, Guatemala, [translated from Spanish], in Stanley, 2017, p. 106)

In good faith, I cited her story when I wrote the book. See, I mused: not all “activism” *is* activism. But then Barbara’s own implied activism began to unravel, too. *Is* this local agency resisting foreign meddling? Or is this a victory for the oppression and injury of workers? After reading more about the short-handled hoe, I have come to understand this story differently from how I first saw it.

The short-handled hoe*—el cortito* (the “shorty”)—was banned in California in 1975, after a series of Industrial Safety Board hearings. In these, evidence was sought from employers, union leaders, farm workers (most of whom were Central American migrants) and the physicians treating the back injuries caused by their bending double to use the short-handled hoe. Evidence against *el cortito* included medical:

When I used the short-handled hoe my head would ache, and my eyes hurt because of the pressure of bending down so long. My back would hurt whenever I stood up or bent over. I moved down the row [of crops] as fast as I could so I could get to the end and rest my back for a moment. (Farm worker, cited by Murray, 1982, p. 29)

I can unequivocally say that the use of this hoe will cause tissue injury and severe back pain, and late may result in degeneration of the intervertebral discs and other supporting elements of the spine, thereby causing pain, limitation of motion, increased vulnerability to severe injury, and in many cases complete physical disability. (Physician, cited by Murray, 1982, p. 34)

But *el cortito*’s effect was not just catastrophic for individuals. Its capacity to injure also caused a rapid turnover of workers, which, in turn, made it difficult to organize trade unions (Murray, 1982, p. 38).

Why, then, was *el cortito* ever used at all? Employers’ testimonies cite a clear advantage: surveillance: “With the long-handled hoe I can’t tell whether they are working or just leaning on their hoes. With the short-handled hoe, I know when they are not working by how often they stand up” (Agricultural supervisor, cited by Murray, 1982, p. 28).

So, *were* the *gringos* who brought the high-quality, long-handled hoes as naïve as Barbara suggests? I don’t think so. I do wonder who, precisely, cut down the handles: was it the workers themselves or was it perhaps their supervisors? This detail was elided from the story, but it is important. The Central American workers themselves, in the California case, certainly did *not* prefer *el cortito*. A Central American agricultural worker from that case, is recorded as saying:

The message was clear: if I didn’t like working with the short-handled hoe, I could quit. There were others to do the work. The contractor didn’t have to deal with what the farm workers wanted, so we continued to use *el cortito*. … They didn’t want the farm worker to stand up. Psychologically, that gives us some dignity. …When you are bent over, it’s showing humility. … It was time for us to overcome these things. (Cited by Murray, 1982, p. 31)

Instead of being a local preference, is *el cortito* more a symbol of laborers’ lowly position and a tool of surveillance by their supervisors? If so, can *el cortito* be located as conceptually adjacent to homophobia, as a local phenomenon for which there is no justification in a universalist model of social justice but as something that can, perhaps, be turned into a local symbol of resistance against what appears to be neocolonial *gringo* meddling?

There is no easy answer. Pitted against one another are, on the one hand, an ethical universalism and its potential for the reinscribing of neocolonial power relations, versus, on the other hand, cultural relativism and the risk that phenomena like homophobia and *el cortito* will thrive thanks to a (misplaced?) postcolonial resistance. Decolonization, on the one hand, and calling out social injustice, on the other, may thus be contradictory. Either may be the purpose of activism, which is why, as a construct, activism is rather empty.

## Academia

Related, how are we to define activism in an academic setting? Farmer (2005), researching structural violence and medical ethics in “developing world” contexts, writes:

“There have been few attempts to ground medical ethics in political economy, history, anthropology, sociology, and the other contextualizing disciplines.” (p. 204)…“The quandary ethics of the individual constitute most of the discussion of medical ethics.…The countless people whose life course is shortened by unequal access to health care are not topics of discussion.” (p. 174)

There is a need, therefore, to go beyond the individualist ethics of medical (mal)practice, which tend to be the focus of academic medical ethics. Instead, we must consider structural violence, including questions of who does, or does not, get to become a patient in the first place. This is a critical medical ethics that concerns itself less with whatever Candace or George are doing to their Central American patients and more with larger-scale social justice.

Farmer’s reframing of medical ethics is an example of theorizing as activism. His thinking goes beyond the immediate “case” and into underlying constructs to understand what is happening at a generalizable level (in the conceptual sense rather than the brutalist generalizability of sample-to-population positivism). Similarly, I critique as structurally violent the existence of “volunteer tourism” within a postcolonial power relationship; this is another example of theorizing as activism.

But as posited above, is there a risk that those of us engaged with academic activism are guilty of precisely the same imperialisms of which we are so roundly critical elsewhere, trammeling young volunteers’ attempts at “helping” within our own critical discourses of systemic change? Perhaps it is necessary for everyone to start somewhere. Perhaps it is even necessary, sometimes, for us to *not* jump to critiquing, but instead to listen to and to hear where others are coming from. Perhaps this text, itself, is paradoxically part of the problem it is critiquing: by roundly critiquing George and Candace, am I perpetuating my own kind of axiological violence? This is another way in which we need to problematize “activism” much more than we already do. (As I hope I am conveying, this is not as simple as it looks.)

Then, in academic activism, there is also the question of reach. Are all my academic words—and yours—subsumed into the white noise of neoliberal productivity in which we churn out more and more to appease the small administrative gods who value less and less (e.g., Mewburn, 2019)? Does any of what we do in the name of academic activism actually change anything? Scottish comedian Frankie Boyle (2015) writes:

Give a man a fish and he can eat for a day. Give him a fishing rod and he can feed himself. Alternatively, don’t poison the fishing waters, abduct his great-grandparents into slavery, then turn up 400 years later on your gap year talking a lot of shite about fish.

Reading this, I crumble. Even as I write academic publications and tell myself I am “doing activism,” a popular entertainer makes a comment that, in four short lines, conveys precisely the point that I am trying, in hundreds of thousands of words, to make. And people hear him. In contrast, Farmer (2005, p. 228) writes that his team “conducted work and published it” but that “research did not figure on the wish list of the people we were trying to serve. Services were what they asked for” Farmer (2005). If even Farmer’s work is not particularly valued by the people on the ground, then what chance is there that I might positively influence Candace or George? All believe they are “doing activism.” Few will ever read my esoteric, academic papers. What good did I do?

Here is what I cling to. After Freire (1986), my activism is *conscientização*, a “consciousness raising” and, I hope, a seed of *conscientização* for others, too. I teach in higher education and it may be that some of my hard-won *conscientização* will leak out into my classes. Perhaps, instead of medical volunteering in Central America (*practicing on* local people, and implicitly holding them in low esteem), my students might choose, instead, to learn Spanish, or weaving, or art, or Mayan cosmology—anything, really—in Central America (thus *learning from* local people, and explicitly holding their culture in much higher esteem). This is my hope.

But I do despair that comedians, such as Frankie Boyle, are the center of cultural gravity in a way that even the most successful academics are not. If my activism is not about improving Nicaraguan clinics and I must rely on those I can influence, would I not be better placed as an influencer outside of academia? I wonder.

## Conclusion

“Activism,” explored here, is difficult to define and thus to measure, and the actions of all the “activists” in this article would be easy to reframe in other ways. Putting untrained U.S.-American teenagers in positions of power, in which they pretend to be doctors for those who cannot afford proper medical care—this could equally well be called human experimentation. Similarly, is Barbara’s resistance false consciousness? And is my own academic activism wishful thinking? Or even axiological violence? This paper has asked at least as many questions as it has answered. I wish it were otherwise, but as I hope to have conveyed, there is a need for complexity here, and the rush toward facile answers is part of the problem.

Decolonization, on the one hand, and addressing social injustice, on the other, are sometimes contradictory, and “activism” needs to address this. This paper has explored some of the issues inherent in who gets to claim they are doing activism and why. It has suggested a framework of structural violence, much bigger than activism itself, that acknowledges intersectional power relations. This draws, perhaps improbably, from the idealism and liberation theology of the original *internacionalistas*: those who went to help the green shoots of the people’s revolutions in Central America in the 1980s, before “helping” was subsumed, a generation later, into the neoliberal voluntourism industry.
